# Tool for genomic selection and breeding to evolutionary adaptation: Development of a 100K single nucleotide polymorphism array for the honey bee

**DOI:** 10.1002/ece3.6357

**Published:** 2020-06-08

**Authors:** Julia C. Jones, Zhipei G. Du, Richard Bernstein, Monique Meyer, Andreas Hoppe, Elmar Schilling, Martin Ableitner, Katrin Juling, Regina Dick, Anja S. Strauss, Kaspar Bienefeld

**Affiliations:** ^1^ Institute for Bee Research Hohen Neuendorf Germany; ^2^ School of Biology and Environmental Science University College Dublin Dublin Ireland; ^3^ Eurofins Genomics Ebersberg Germany

**Keywords:** adaptation, breeding, genomic selection, honey bee, single nucleotide polymorphism chip, *Varroa* resistance

## Abstract

High‐throughput high‐density genotyping arrays continue to be a fast, accurate, and cost‐effective method for genotyping thousands of polymorphisms in high numbers of individuals. Here, we have developed a new high‐density SNP genotyping array (103,270 SNPs) for honey bees, one of the most ecologically and economically important pollinators worldwide. SNPs were detected by conducting whole‐genome resequencing of 61 honey bee drones (haploid males) from throughout Europe. Selection of SNPs for the chip was done in multiple steps using several criteria. The majority of SNPs were selected based on their location within known candidate regions or genes underlying a range of honey bee traits, including hygienic behavior against pathogens, foraging, and subspecies. Additionally, markers from a GWAS of hygienic behavior against the major honey bee parasite *Varroa destructor* were brought over. The chip also includes SNPs associated with each of three major breeding objectives—honey yield, gentleness, and *Varroa* resistance. We validated the chip and make recommendations for its use by determining error rates in repeat genotypings, examining the genotyping performance of different tissues, and by testing how well different sample types represent the queen's genotype. The latter is a key test because it is highly beneficial to be able to determine the queen's genotype by nonlethal means. The array is now publicly available and we suggest it will be a useful tool in genomic selection and honey bee breeding, as well as for GWAS of different traits, and for population genomic, adaptation, and conservation questions.

## Graphical abstract

The European honey bee (*Apis mellifera*) is one of the most ecologically and economically important pollinators worldwide, yet to date a high‐throughput, high‐density genotyping array has not been available for the bees. Here, we developed the first high‐density chip for honey bees with probes for over 100,000 SNPs. SNPs were detected by conducting whole genome resequencing of bees from throughout Europe, and the selection of SNPs for the chip was done in multiple steps using several criteria. Photo by Clement Tang.

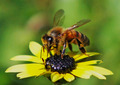

## 1 INTRODUCTION

The European honey bee (*Apis mellifera*) is one of the most ecologically and economically important pollinators. It is the most frequent floral visitor in natural habitats worldwide (Hung, Kingston, Albrecht, Holway, & Kohn, 2018), and it is a key contributor to pollination services in agriculture where there is an overall value of €153 billion globally (Gallai, Salles, Settele, & Vaissière, 2009). Additionally, honey bees have been managed for thousands of years as a source of honey and beeswax. Thus, it is not surprising that the genome of the honey bee was among the first to be sequenced (Weinstock et al., 2006), and that honey bees have been the focus of a diverse array of studies on ecology, the evolution of sociality, the genetic basis of behavior, and population genomics (e.g., Hung et al., 2018; Seeley, 1985; Shpigler et al., 2017; Smith, Toth, Suarez, & Robinson, 2008; Wallberg et al., 2014). Importantly, in recent decades honey bee colony numbers have reportedly declined in some areas with the most likely explanation being a combination of stressors, including synergistic interactions between parasites, pathogens, toxins including pesticides, and other stressors (Alburaki et al., 2015; Genersch et al., 2010; Goulson, Nicholls, Botías, & Rotheray, 2015; Sanchez‐Bayo et al., 2016; Sánchez‐Bayo & Wyckhuys, 2019). Despite the obvious importance of the ecosystem services they provide, broad focus in the scientific community, and economic value, one key genomic tool, a high‐throughput, high‐density genotyping array, is not yet available for screening genome‐wide polymorphic variation in honey bees.

High‐density single nucleotide polymorphism (SNP) arrays, or “SNP chips” (Spencer, Su, Donnelly, & Marchini, 2009; Syvanen, 2001), are a fast, accurate, and efficient technique for genotyping thousands of polymorphisms in high numbers of individuals. SNP chips are one of the main technologies appropriate for analytical techniques that require large sample sizes (Kim et al., 2018), such as genome‐wide association studies (GWAS), quantitative trait locus (QTL) linkage mapping (Spötter, Gupta, Mayer, Reinsch, & Bienefeld, 2016; Visscher et al., 2017), molecular quantitative genetics including genomic selection in breeding programs (Gienapp et al., 2017; Jensen, Szulkin, & Slate, 2014), and studies utilizing relatedness/inbreeding coefficients (Powell, Visscher, & Goddard, 2010). The development of SNP chips has become routine in human genetics and in farmed animal and plant breeding, where genomic selection has revolutionized breeding. Traditionally, animal breeding, that is, selective breeding for economically important traits such as milk yield, was based on phenotypic records, where individual records and those of relatives were combined to estimate breeding values (Meuwissen, Hayes, & Goddard, 2016). Genotyping arrays can facilitate rapid and more accurate breeding value predictions (or genomic breeding value estimations) (Gienapp et al., 2017; Meuwissen, Hayes, & Goddard, 2001; Meuwissen et al., 2016). Genomic selection was first applied, and is now widely used, in cattle (Hayes, Bowman, Chamberlain, & Goddard, 2009). In Atlantic salmon, breeding programs using genomic selection have been suggested to have the potential to increase selection accuracy, genetic gain, and reduce inbreeding (Houston et al., 2014). In plant breeding, the development of SNP genotyping arrays for germplasm characterization is now common, with many crop plant species, including wheat, rice, maize, potato, rapeseed, apple, and tomato, having their own custom SNP genotyping arrays (Bayer et al., 2017; Bianco et al., 2016; Chen et al., 2014; Clarke et al., 2016; Ganal et al., 2011; Mason et al., 2017; Sim et al., 2012; Vos, Uitdewilligen, Voorrips, Visser, & van Eck, 2015; Winfield et al., 2016).

To date, SNP chips are less widely used in ecological and evolutionary research and in wild populations, but are gaining impetus. For instance, a high‐density chip (500,000 SNPs) was recently developed for performing genomic studies of great tit (*Parus major*) populations, and this chip has been used to study the genetic architecture of exploration behavior (Kim et al., 2018). Other examples using smaller chips (40–50K) in wild vertebrate populations include Soay sheep (*Ovis aries*) (Johnston et al., 2013), collared flycatchers (*Ficedula albicollis*) (Kawakami et al., 2014; Silva et al., 2017), and house sparrows (*Passer domesticus*) (Silva et al., 2017). A SNP chip has also been developed for the dengue and yellow fever mosquito, *Aedes aegypti*. This mosquito chip has applications in a range of research questions, from studies of potential range shifts due to climate change, to characterizing the genetic underpinnings of disease transmission (Evans et al., 2015).

The development of a high‐density SNP chip for honey bees provides a cost‐effective, high‐throughput option for screening thousands of SNPs, and is also a tool that can be applied to diverse research questions. More specific SNP assays have already been developed and applied in honey bees, including a 44K SNP assay focusing on the analysis of defense behavior against one of the most destructive honey bee parasites—the *Varroa destructor* mite which transmits several viral diseases (Spötter, Gupta, Nurnberg, Reinsch, & Bienefeld, 2012). SNP panels have also been developed to detect Africanized honey bees (Chapman et al., 2015, 2017). Further, a SNP panel was developed for molecular identification and the estimation of introgression levels of the “C” honey bee lineage, with the threatened subspecies *A. mellifera mellifera* (Henriques et al., 2018). A high‐density SNP chip can extend such studies by enabling the detection of polymorphisms related to defenses against other pathogens and diseases, and potentially also the estimation of genetic differentiation of other honey bee subspecies and populations. Dense genome‐wide SNP data can facilitate fast and accurate honey bee breeding programs, GWAS of diverse traits, population genomic, adaptation and conservation questions, and investigations of the genetic underpinnings of disease resistance and resistance to other stressors.

To this end, we developed a high‐density Illumina Custom Infinium Genotyping chip for honey bees with probes for over 100 000 SNPs based on the subspecies *A. mellifera carnica and A. mellifera mellifera*. We combined the wealth of available genomic information and resources for the honey bee and several selection criteria to detect and select genome‐wide SNPs applicable to a diversity of key traits in bee breeding, and in evolutionary and ecological studies. Additionally, we test the genotyping success of different tissue types that can be used as alternatives to sacrificing the queen of a colony—a key development for honey bee breeding and genomic selection studies.

## 2 MATERIALS AND METHODS

### 2.1 Samples and DNA sequencing

To identify SNPs to include on the chip whole‐genome resequencing was performed on 61 unrelated honey bee drones. Samples were sourced from breeders of the international *A. mellifera carnica* breeding program (37 samples from Germany and Austria), the international *A. mellifera mellifera* breeding program (12 samples from Switzerland, Austria and Norway), and the protected *A. mellifera carnica* conservation region Slovenia (12 samples) (Table S1). Thus, 49 of the drones were from the subspecies *A. mellifera carnica*, and 12 from the subspecies *A. mellifera mellifera*. The subspecies of a sample bee was guaranteed by quality control of the breeding and conservation programs, and additionally confirmed with the use of morphometric traits known to distinguish the two subspecies (Ruttner, 1988; Tiesler, Bienefeld, & Büchler, 2016). DNA was extracted from the flight muscles of each individual drone using the NucleoSpin^®^ 8 Food (Macherey‐Nagel) (Eurofins Ebersberg). Genomic libraries were prepared at Eurofins Ebersberg, using the NEBNext Ultra DNA Library Prep Kit E7370 (New England Biolabs). Pools of three individually barcoded samples were sequenced on an Illumina HiSeq 2500 platform at Eurofins. Sequencing was paired‐end, 2 × 100 bp, using HiSeq Flow Cell v3 and TruSeq SBS Kit v3. Details of the coverage for each drone sample are presented in Table S1.

### 2.2 SNP detection

A total of 6,231,233,743 honey bee sequence reads (average of 102,151,372.8 reads per sample, *SD* = 26 875 640.2) were mapped to the reference genome Amel_4.5 (INSDC assembly GCA_000002195.1). We identified and marked PCR duplicates using Picard Tools MarkDuplicates (version 1.108, http://picard.sourceforge.net). Only reads with a minimum alignment quality of 1 (Phred quality score) were used for variant detection, meaning all reads with ambiguous alignments were excluded. Variants were called using VarScan (version 2.3, (Koboldt et al., 2012)). VarScan was used to process the mapping output one base at a time and compute the number of bases supporting each observed allele (minimum read depth 5). Only bases meeting the minimum base quality of 15 (Phred quality score) were considered. Using VarScan each observed allele was examined and tested for support by at least two reads, and a minimum allele frequency threshold of 1%.

Primary filters were also applied to all detected SNPs prior to the SNP selection steps. Specifically, a filter was applied to remove any SNPs with unknown bases within a flanking area of 50 bp on either side of the SNP, and any loci with possible indels or multi‐allelic SNPs were removed.

### 2.3 SNP selection

**FIGURE 1:**
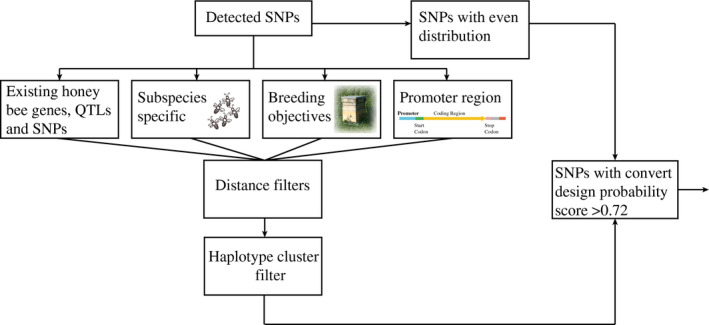
Summary of SNP selection workflow steps for development of the HDHB SNP chip. SNP numbers for each criterium are provided in Table 1

During SNP detection, many more SNPs were identified than could be included on the chip. Therefore, to prioritize which SNPs to use on the chip, selection was done in multiple steps using several criteria (see also a summary of the SNP selection pipeline in Figure 1):

#### 2.3.1 Known honey bee genes, QTLs, and SNPs

A list of candidate genomic regions including genes, QTLs, and SNPs that may be involved in general honey bee biology, and/or could potentially explain variation in a range of honey bee traits, was compiled, and the location of the detected SNPs within these regions (specifically within the candidate genes) was determined using a custom Python script. The list of candidate regions was compiled through searching the existing literature, using keyword searches of genes in the NCBI database, selecting genes in signaling, and immune pathways (including Notch, JAK‐STAT and Toll), and including all putative genes reported in the Beebase project (Elsik et al., 2014). Included in candidate SNPs brought over from existing literature were a large number of SNPs previously identified as being associated with the detection and uncapping of *Varroa*‐parasitized brood in a GWAS (Spötter et al., 2016). This GWAS study utilized a 44K SNP chip designed for the analysis of hygienic behavior of individual worker bees against *V. destructor* (Spötter et al., 2012). Here, we included 498 SNPs found to be significantly associated with hygienic behavior (Spötter et al., 2016; A. Spötter, P. Gupta, M. Mayer, N. Reinsch, & K. Bienefeld, unpublished data).

To further prioritize the above SNPs for use on the chip, we implemented genomic region (coding) and distance criteria (see below) where SNPs within coding regions and with greater estimated regulatory effects were selected at higher density. Different distance criteria (in addition to genomic region criteria) were tested, and distance criteria predicted to enable a final SNP chip density of ~100,000 SNPs were implemented. The predicted effects of all SNPs were determined using SnpEff (version 4.3) (Cingolani et al., 2012). A customized Python script was used to select SNPs with an even distribution throughout the genome. Additionally, the distribution of genes selected from the literature also influenced the distribution of SNPs throughout the genome. The minimum distance allowed between SNPs within coding regions was 300 bp; the distance between SNPs located outside of coding regions but estimated to have a high to moderate effect was 1,000 bp, and a low effect was 5,000 bp; the distance between SNPs located within the putative genes reported in Beebase was 3,000 bp. Additionally, the minimum distance allowed between SNPs located within promoter regions was 500 bp. Promoter regions were identified by characteristic sequence pattern matching near coding regions. The sequence patterns used were TATA box and BRE combinations 25–35 bp upstream of the coding area, and the promoter region was then defined upstream of these markers. Specifically, when both these patterns were matched, the promoter region was defined as being within 200bp upstream of the beginning of the TATA box.

#### 2.3.2 Subspecies and breeding objectives

All detected SNPs associated with subspecies or specific breeding objectives were also selected at higher density. Subspecies association (carnica/mellifera) was estimated for all detected SNPs. For each SNP and each pair of bases, a significant association with subspecies was estimated using a *t* test. The distance allowed between SNPs associated with subspecies was 500 bp.

The association between all detected SNPs and three breeding objectives ([a] honey yield, [b] gentleness, and [c] *Varroa* resistance) was also estimated. This was achieved using 37 of the drones that were from known breeding queens of the *A. mellifera carnica* breeding program and were therefore associated with the breeding values of the 2015' breeding value estimation in www.beebreed.eu. A *t* test was performed to determine whether the average breeding values differed significantly between bases (*p*‐value < .01). No correction for false positives was applied in this test because as this was a filtering step, emphasis was on sensitivity not specificity. The distance allowed between SNPs associated with breeding objectives was 100 bp.

#### 2.3.3 Haplotype filter

To further reduce the number of detected SNPs for use on the chip, all SNPs selected under the above criteria were additionally subjected to a haplotype filter (cluster filter). That is, out of any linked SNPs, only one SNP was selected. Loci (SNPs) were designated as belonging to the same cluster if their vectors of bases (i.e., all bases) for all 61 sequenced drones were identical. It was assumed that the drones were representative of the population, and that different loci in one cluster are redundant (as markers), and therefore the SNPs, except one within a cluster, are obsolete (i.e., where including more SNPs would not add any further marker information). Single SNPs from each cluster were chosen by selecting the first SNP in the cluster that had a distance of >300 bp to already selected neighboring SNPs. As above, the 300 bp distance was chosen under the objective of developing a final SNP chip with approximately 100,000 SNPs.

#### 2.3.4 Illumina SNP marker score cutoff

All SNPs remaining after implementing the above selection criteria were subject to the further criterion of how likely it was that they could be converted to a working scorable assay on the chip. The list of SNPs and their flanking sequences were uploaded to the MyIllumina online portal (https://dashboard.my.illumina.com) and design scores were calculated. Design scores represent the probability that the probe will be successful in a genotyping assay. Lower probability scores can be caused by the possibility of secondary structures forming, or if a marker is in a duplicated or repetitive region. SNPs with a convert design score >0.72 were retained for inclusion on the chip. This threshold is even more stringent than those used in the design of HD Axiom chips for chicken, catfish, water buffalo, and great tits where thresholds of 0.20, 0.50, 0.60, and 0.69 were used, respectively (Iamartino et al., 2017; Kim et al., 2018; Kranis et al., 2013; Liu et al., 2014). In total, 103,270 SNPs were included on the chip.

### 2.4 SNP validation and tests of tissue performance

To date, genotyping honey bee queens usually involves sacrificing the queen. However, honey bee biology research, especially in bee breeding, benefits from determining the queen's genotype by nonlethal means. Therefore, here we tested the DNA genotyping call rate success of different tissue types that could be used in place of the queen (queen cells where the queen develops, drone eggs, drone larvae and drone pupae). Additionally, we tested how well different sample types from a colony (queen cells and different numbers of drone eggs) represent the queen's genotype using empirical data, that is, where the queen's genotype is inferred from her haploid drone offspring (eggs). Discrepancies in genotype representation of the queen can arise due to queen cells potentially being contaminated by worker DNA, as for instance, workers enter the cells to feed the developing queens, and because drones are haploid successfully determining the queen's genotype using drone samples relies on using a sufficient sample size of drones.

#### 2.4.1 Sample collection

*Apis mellifera carnica* queens, queen cells, drone pupae, larvae or eggs were collected by selected honey bee breeders in Germany, Austria, the Netherlands, and Switzerland (beebreed.eu) and sent to the authors at the Institute for Bee Research, Hohen Neuendorf. A large resource of samples was collected in this way, and their DNA was extracted and genotyped (as per the methods described below). In addition to forming the basis for setting the call rate thresholds (see below), these data will be utilized in future analyses. For all other analyses conducted here, subsets of the genotyping data were used.

#### 2.4.2 DNA extraction

Honey bee samples were sent to Eurofins Ebersberg, for DNA extraction. DNA was extracted from 3,272 samples, including individual and colony replicates. Samples included replicates of the same individual where different tissues were used for DNA extraction that is, queen and queen cell (see below); and pairs of individuals from the same colony with known relationships, for example, queen–daughter. A summary of the individual unique colonies from which samples were taken is available on Dryad (Table S2, https://doi.org/10.5061/dryad.gxd2547gp).

DNA was extracted from queen cells of recently emerged queens using a modified version of the DNeasy Blood and Tissue Kit (Qiagen), for nails, hair or feathers). Frozen queen cells were incubated for 15 min in xylene, washed in ethanol (99%), dried at 37°C, cut into small pieces, and then the manufacturer instructions were followed (for further details see Figure S1). DNA was extracted from queen flight muscles and 30–120 drone eggs using the KingFisher automated extraction method (Thermofisher) and MagSi‐DNA allround Kit (Magtivio). DNA of pools of 30–60 adult drones and pools of ca. 60 larvae or pupae was extracted in two subsamples (1 g of homogenized tissue per sample) using the Maxwell automated extraction system (Promega) and the Maxwell‐Kit (16 FFS Nucleic Acid Extraction System, Custom (REF.: X9431, Promega)). DNA subsamples were pooled after extraction.

#### 2.4.3 Genotyping and call rate thresholds

Genomic DNA was sent to Eurofins Genomics in Aarhus, Denmark and genotyped in batches of 96. As a first step, 400 samples, including all of the different tissue types, were selected for cluster file generation. The raw data were analyzed using the Illumina GenomeStudio software v 2.0 with standard settings and custom cluster generation. The 200 best performing samples were selected (based on a call rate >98%) to optimize the custom cluster file. The cluster positions were then manually checked using the GenomeStudio software, and 3,637 markers were removed due to bad performance based on manual inspection of the clusters. The 5,000 lowest scoring (GenTrain score) markers were manually adjusted. Cluster positions were then exported, and these positions were used in all subsequent genotyping analyses.

Genotyping quality thresholds were set by examining markers (SNP) and samples carried forward under different SNP call rate thresholds (80%, 90%, 95% and 97% thresholds). A balance was found between retaining the highest number of SNP markers, while also retaining a high number of successfully genotyped samples. To set the quality control thresholds, all genotyped samples were included in the data examination (including different individuals from the same colonies and repeat genotypings of the same samples). First, we set a marker quality threshold of call rate >90%, meaning all markers that were called in <90% of samples were removed. Second, we set a sample quality threshold of call rate >90%, meaning all samples where <90% of all remaining markers were called were removed.

#### 2.4.4 SNP statistics for a sample population

For each SNP that passed the marker quality threshold, the minor allele frequency (MAF), Hardy–Weinberg equilibrium (HWE) (*p*‐values of chi‐square tests), and call rates were calculated using a set of quality‐controlled samples (*n* = 2,734). All 103,270 SNPs selected for the chip were considered for these analyses, and included 6,386 SNPs of unplaced contig origin, as well as 580 unmapped SNPs. MAF and HWE for each locus were calculated using the GenomeStudio 2.0 software according to the GenomeStudio Framework User Guide (Illumina) with default parameters.

#### 2.4.5 Quantifying genotyping error rate

A total of 61 replicated samples (i.e., where the same DNA had been genotyped twice) (marker quality >90% call rate; sample quality >90% call rate) enabled an estimation of the number of inconsistent genotype calls between different genotyping attempts of the same bee. This was achieved by calculating the average of different calls between replicates over all SNPs (i.e., where both samples had genotype calls at that locus, but the calls were different).

#### 2.4.6 Tissue performance tests

To determine the SNP chip genotyping success rate of different tissue types, we measured the average genotyping call rate (i.e., success of being called at all) of all genotyped samples (see sample numbers in Table 2) and compared them using an ANOVA, with tissue type as a factor and call rate as the response variable, and Tukey post hoc tests in R.

#### 2.4.7 Representation of the queen's genotype

To determine how well queen cells and pools of drone eggs represent the queen's genotype, we compared the percentage of different calls with the queen (where DNA had been extracted from the queen's flight muscle) and these samples (i.e., queen cells, pools of 30 drone eggs and pools of 120 drone eggs [minimum number of 30 drone eggs determined using mathematical modeling {Bernstein et al., in prep.}]). For this comparison, we used samples that enabled the highest possible call rates for both groups (97%). Results were compared using an ANOVA in R, with tissue type as a factor and percentage of different calls as the response variable.

## 3 RESULTS

### 3.1 SNP detection and selection

To detect candidate SNPs for inclusion on an Illumina Infinium high‐density SNP chip for honey bees (*A. mellifera*) (High‐density honey bee SNP chip: HDHB‐chip), whole‐genome resequencing of 61 unrelated honey bee drones from throughout Europe was completed. SNP selection was subsequently carried out in multiple steps using several criteria (see methods and Figure 1). Alignment of the genome sequence data to the honey bee reference genome (Amel_4.5) enabled the identification of 4,970,950 putative variable SNP positions (total SNPs before filtering and selection). After quality filtering and the implementation of the SNP selection criteria, a total of 103,270 SNPs were included on the final chip.

**FIGURE 2:**
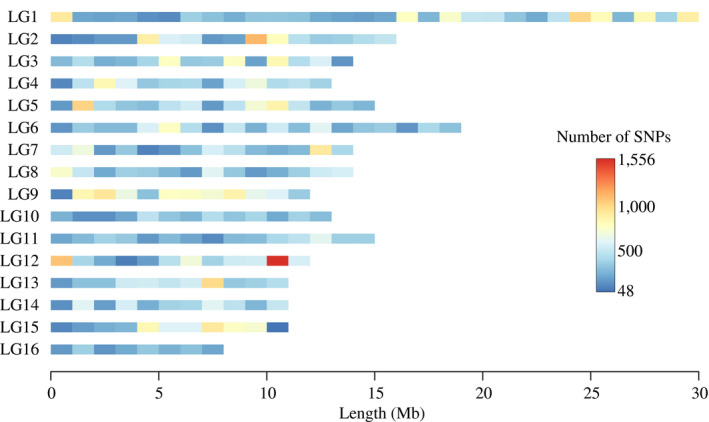
Genomic distribution of SNPs included on the HDHB SNP chip

SNPs were selected by searching the existing literature, conducting keyword searches of the NCBI database, and selecting genes in key signaling and immune pathways. Genomic region and distance criteria were also implemented and SNPs associated with *A. mellifera carnica* and *A. mellifera mellifera* subspecies and breeding objectives were selected. Finally, an Illumina marker score cutoff (convert design score >0.72) was implemented. A summary of the SNPs selected under the different selection criteria is provided in Table 1. A detailed overview of the candidate genomic regions selected from the literature, keyword searches of genes in the NCBI database, and genes in key signaling and immune pathways is provided in Tables S3–S5. The genomic distribution of SNPs included on the chip can be visualized in Figure 2. The SNP chip is available from Eurofins Genomics (https://www.eurofinsgenomics.eu/en/eurofins‐genomics/service‐corner/).

**TABLE 1:** Number of SNPs by SNP selection criteria

SNP selection criteria	Number of SNPs
Existing literature	53,789
*Varroa‐*resistant behavior, including overlap other categories	1,477
Subspecies specific, including overlap existing literature	12,964
Breeding objectives: honey yield, gentleness, *Varroa* resistance	14,304
Promoter region	2,035
Haplotype cluster	46
Within genes, and with moderate–high predicted effects	5,400
Others	13,255

### 3.2 SNP validation and tests of tissue performance

#### 3.2.1 Genotyping error rate and tissue performance tests

The average percentage of different calls between replicate genotypings of the same bees (61) over all SNPs was 0.23%. Additionally, we find that as expected, DNA extracted from queen flight muscle tissue enables the highest average call rate (98%) of all tissue types tested, and is significantly higher than the average call rate obtained from queen cell (83%), pupae (94%), larvae (95%), and egg tissues (96%) (Tukey post hoc tests, *p* < .05). All tissues provide average genotyping call rates above 90%, apart from queen cells where the average call rate (83%) is below 90% (Table 2).

#### 3.2.2 SNP statistics for a sample population

The newly constructed SNP chip was used to evaluate call rate, MAF and HWE, in 2,734 samples from unique colonies (Table S6). An average MAF of 17.6% was found across the 2,734 samples after excluding SNPs meeting one of the following criteria: (a) call rate < 90%; (b) significant deviation from HWE after Bonferroni correction (chi‐square *p*‐value < .05 × 10^5^); c) and MAF < 5%. A large proportion of SNPs (41%) were found to have a MAF < 5%. Under a milder threshold of a MAF 1%, and when call rate and HWE estimates are controlled for as above, we find that 27% of SNPs would need to be removed from analyses, and that the resulting average MAF is 14.8%.

**TABLE 2:** Average call rates achieved using different tissue types

Tissue type	Average call rate (%) ± *SE* (same scale)	Number of samples with call rate > 90%	Total number of samples
Queen flight muscles	98 ± 6	840	907
Queens cells	83 ± 17	84	175
Drone pupae	94 ± 6	45	62
Drone larvae	95 ± 6	59	75
Drone eggs	96 ± 7	1,666	1,860

#### 3.2.3 Queen representation

We find no significant difference in how well the queen's genotype is represented between the alternative tissue types (*F*(2,30) = 6.11, *p* = .55). The queen cells and all drone egg pools tested (30 eggs, and 120 eggs) represent the queen's genotype well overall (percent different calls between the queen's genotype and pools of drone eggs of the same colony are shown in Table 3). Tissue from queen cells where queens had recently emerged from had the lowest percentage of different calls compared with the queen's genotype (from flight muscle tissue) (Table 3). Additionally, the queen's genotype was slightly more accurately represented when a higher number of drones from the colony were pooled and genotyped.

**TABLE 3:** Representation of the queen by queen cell and drone egg tissues

Tissue comparison	Pairs of samples	Different calls (% of all loci) ± *SE*	Queen flight muscle call rate ± *SE*	Call rate of the alternative tissue ± *SE*
Queen versus queen cell	19	0.09 ± 0.20	99.42 ± 0.60	99.31 ± 0.61
Queen versus 120 eggs	7	0.14 ± 0.12	98.72 ± 1.07	98.71 ± 0.67
Queen versus 30 eggs	7	0.17 ± 0.14	98.52 ± 1.02	98.06 ± 0.52

Note

All queen genotypes were generated using DNA from flight muscle tissue in these comparisons.

## 4 DISCUSSION

In this study, we describe the development of a high‐density SNP chip for the honey bee *A. mellifera* (HDHB‐chip) and test error rates of the chip, the performance of different tissues in genotyping, and how well alternative samples represent the queen's genotype. We show that most SNPs on the chip could be genotyped accurately in repeat genotypings, with an average error rate of <1%. Similar and higher density chips are commonly used in agriculturally important species (Rincon, Weber, Eenennaam, Golden, & Medrano, 2011; Winfield et al., 2016), in human studies (International HapMap Consortium et al., 2007; Simonson et al., 2010), model organisms (Yang et al., 2011), and companion animals (Hayward et al., 2016). Such tools in wild populations are less common but are increasingly being developed (Evans et al., 2015; Johnston et al., 2013; Kawakami et al., 2014; Kim et al., 2018; Silva et al., 2017). This is the first time that a robust high‐density SNP chip has been made available for the honey bee. We suggest that this chip will be a useful tool in studies spanning a variety of research areas, from applications in agriculture and breeding programs, to population genetics and adaptive evolution questions.

For analyses requiring large sample sizes, the main alternative technologies to whole‐genome sequencing are SNP chips, (Spencer et al., 2009; Syvanen, 2001) and genotyping‐by‐sequencing (GBS) methods (Davey et al., 2011; Elshire et al., 2011) such as restriction‐site associated DNA sequencing (RAD‐seq) (e.g., Franchini, Monné Parera, Kautt, & Meyer, 2017; Heliconius Genome Consortium, 2012; Hohenlohe et al., 2010; Jones, Fan, Franchini, Schartl, & Meyer, 2013). Whole‐genome sequencing approaches can still be prohibitively expensive for many research groups and applications (Kim et al., 2018), including for organisms with small genomes such as the honey bee. Further, whole‐genome sequencing requires a greater amount and higher quality of DNA than other approaches, which can be limited when working with some organisms such as insects, and can translate into analyses of large samples sizes being more labor intensive. Although SNP chips tend to be more expensive than GBS approaches, specific target SNPs of interest can be included on a chip whereas marker sites are usually not known before genotyping is conducted under GBS approaches. Additionally, SNP chips tend to have higher call rates per SNP than in GBS approaches, and when using SNP chips, the same SNPs are genotyped in every individual which is not possible with GBS methods (Bajgain, Rouse, & Anderson, 2016). Ascertainment bias is a disadvantage of SNP chip data and can be introduced when SNPs are selected from a small panel of individuals (Albrechtsen, Nielsen, & Nielsen, 2010). Using small panels means there is a higher chance of finding a common SNP with a higher minor allele frequency (MAF) than finding a SNP of low MAF (Albrechtsen et al., 2010). Similarly, there is often a bias toward including SNPs with a higher MAF due to the fact that SNPs have to be discovered before being included on a chip (Kim et al., 2018). However, this is not necessarily a disadvantage for some types of analyses such as GWAS (Kim et al., 2018). Here, the average MAF estimated from 2,734 bees collected by honey bee breeders across Germany, Austria, the Netherlands, and Switzerland (beebreed.eu) (17.6%) is low compared with estimates in other studies utilizing SNP chips to study agriculturally important species such as goats (MAF 25%) (Lashmar, Visser, & Van Marle‐Köster, 2015), horses (MAF range 18%–23%) (McCue & Michelson, 2013), and different cattle breeds (MAF range 17%–22%) (Qwabe, vanMarle‐Koster, Maiwashe, & Muchadeyi, 2013). Although the MAF is relatively low, 73% of SNPs can still be retained under the commonly used MAF threshold of 1%. Ultimately, the best method for genotyping large numbers of individuals depends on the research question, the laboratory, and bioinformatics experience of the user and the funding available (see also Kim et al., 2018).

The majority of SNPs selected for the honey bee chip fall within an extensive list of candidate genomic regions from previous research, including genes suggested to be important in general honey bee biology and/or associated with a range of honey bee traits, and genes in key signaling and immune pathways. The chip includes 498 SNPs found to be significantly associated with the trait of hygienic behavior against the major honey bee parasite, *Varroa* (detecting and uncapping of Varroa‐parasitized brood) in a GWAS (Spötter et al., 2016; Spötter et al., 2012, Spötter et al. unpublished data). Further, a large number of SNPs found to be associated with two of the honey bee subspecies (*A. mellifera carnica* and *A. mellifera mellifera*) (7,680) are also included on the chip, and genomic regions related to subspecies were also mined from the literature and SNPs within these regions were included on the chip. We focused on differentiating these two particular subspecies because *A. mellifera carnica* is the most widely employed subspecies in Eastern Central Europe, and *A. mellifera mellifera* is native to this region and still present (De la Rúa, Jaffé, Dall'Olio, Muñoz, & Serrano, 2009). We suggest that this chip will be a useful tool in studies that require the delineation of these two honey bee subspecies, and potentially additionally for distinguishing the other honey bee subspecies, and in determining the molecular underpinnings of key subspecies traits. Further, a large number of SNPs found to be associated with each of three major breeding objectives (honey yield, 4,791; gentleness, 1,035; and *Varroa* resistance 8,478) used in the beebreed database by breeders across Europe, were also included on the chip. The latter results should be taken as preliminary as the sample size for the analysis for selecting these SNPs was necessarily limited to 37 of the whole‐genome resequenced drones. However, the chip will likely also be particularly useful in bee breeding programs and genomic selection for traits of interest in honey bee breeding in future. We note that because we selected specific SNPs for the chip, the SNPs are not evenly distributed across chromosomes. However, biases in the genomic distribution of SNPs associated with different traits will be interesting to investigate in future studies.

### 4.1 Tests of tissue performance and queen representation

As expected, queen flight muscle enabled the highest average call rate, dependent on higher quality, and quantity of DNA yield (DNA was measured using gel electrophoresis before genotyping). Therefore, the best results will be achieved for population genomics and ecology research questions utilizing flight muscle tissue of honey bee samples. However, important for honey bee breeding and genomic selection questions, we find that although drone eggs, pupae, and larvae enable significantly lower average call rates, they are useful alternative tissue types for DNA extraction and genotyping as they avoid sacrificing the queen and the average call rates for these tissues are above the 90% threshold determined.

Drone eggs (when pools of 30 eggs and higher are used) are also found to be a good alternative to sacrificing the queen while still accurately representing the queen's genotype. Our comparison of the percentage of different genotype calls shows pools of 30 or 120 drone eggs represent the queen's genotype accurately. Thus, overall, we find that using a minimum pool of 30 drone eggs is a reasonable alternative to sacrificing the queen, while still accurately representing the queen's genotype. Further, using 30 drone eggs as opposed to 120 facilitates simpler and more efficient methods because sampling and extracting DNA from a lower number of drone eggs is easier and less time‐consuming. Of note is that when a call rate above 90% is achieved for genotyping queen cell DNA, this tissue provides the most accurate representation of the queen's genotype (a call rate above 90% was achieved in 79% of queen cell samples). Using DNA from queen cells of newly emerged queens is the most accurate possible alternative to sacrificing the queen, and allows immediate breeding value estimation for selection of queens in breeding studies and programs. We suggest that the continued rapid optimization of extracting good‐quality DNA from small amounts of tissue means queen cells are also a good alternative to sacrificing the queen.

High‐density SNP chips continue to offer a robust and relatively straightforward means of investigating different research questions, from genomic selection and breeding topics, to a range of evolutionary genomics and adaptation questions. Eventually whole‐genome sequencing will be used in place of SNP chips, but SNP chips are likely to be used for several more years due to the relatively simple laboratory and bioinformatics protocols and pipelines required, the more affordable costs for investigating population level numbers of individuals, and population genetic questions. We suggest that the methods of development and validation presented here for the honey bee SNP chip will be useful to other researchers developing chips for their study organisms, and that the chip will be a useful tool in a diversity of bee research studies and applications. The honey bee chip we describe is available to other users at Eurofins Genomics.

## CONFLICT OF INTEREST

We declare all authors have no conflicts of interest.

## AUTHOR CONTRIBUTION

**Julia C. Jones:** Conceptualization (equal); formal analysis (equal); investigation (equal); supervision (supporting); writing‐original draft (lead); writing‐review & editing (lead). **Zhipei G. Du:** Conceptualization (equal); data curation (equal); formal analysis (equal); investigation (equal); writing‐original draft (supporting); writing‐review & editing (supporting). **Richard Bernstein:** Data curation (equal); formal analysis (equal); investigation (equal); writing‐original draft (supporting); writing‐review & editing (supporting). **Monique Meyer:** Data curation (equal); investigation (equal). **Andreas Hoppe:** Formal analysis (supporting); writing‐original draft (supporting); writing‐review & editing (supporting). **Elmar Schilling:** Data curation (equal); formal analysis (equal). **Martin Ableitner:** Data curation (equal); formal analysis (equal). **Katrin Juling:** Data curation (equal); formal analysis (equal). **Regina Dick:** Supervision (equal); writing‐original draft (supporting); writing‐review & editing (supporting). **Anja S. Strauss:** Conceptualization (equal); data curation (equal); formal analysis (equal); investigation (equal); supervision (supporting); writing‐original draft (supporting); writing‐review & editing (supporting). **Kaspar Bienefeld:** Conceptualization (lead); funding acquisition (lead); supervision (lead); writing‐original draft (supporting); writing‐review & editing (supporting).

## Supporting information

Appendix S1

## Data Availability

Resequenced drone genomes are reported on the NCBI sequence read archive (PRJNA596071). All SNPs included on the chip are reported on Dryad, as well as the Table summarizing all individual unique colonies from which samples were taken (https://doi.org/10.5061/dryad.gxd2547gp).
