# Supplementary material for: Tool for genomic selection and breeding to evolutionary adaptation: Development of a 100K single nucleotide polymorphism array for the honey bee
Source: Ecol Evol. 2020 Jun 8;10(13):6246–56. doi: 10.1002/ece3.6357 (PMC7381592; doi:10.1002/ece3.6357)
Supplement: Supplementary file 1 — Appendix S1 [file ECE3-10-6246-s001.pdf]

Appendix 1 - Jones et al.

Figure S1

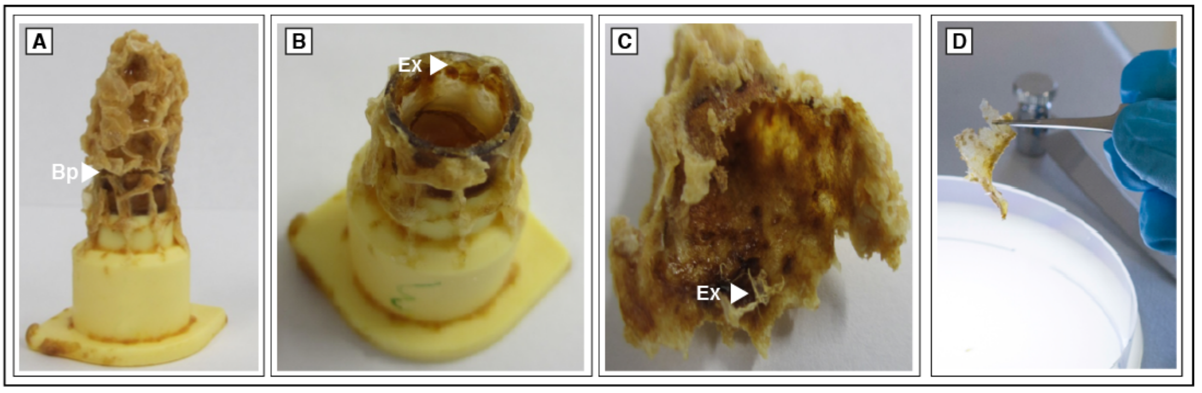

Figure S1. DNA extraction from queen cells. Queen cell connected to a feeding bowl (A). Bp indicates position where queen cells (frozen -20°C) were broken and removed from the feeding bowl. Exuvia (Ex) was immediately removed from the food bowl (B). Exuvia was also removed from the wax cell (C), but the wax cell was first incubated for 15 min in 4 ml of xylene at RT. Isolated exuviae from both the food bowl and wax cell (D) were washed twice in ethanol (99%), dried at 37°C and cut into smaller pieces. Total DNA was extracted using the the DNeasy® Blood & Tissue Kit according to the manufacturers' instructions with an overnight lysis step.

Appendix 1 - Jones et al.

Table S1. Sixty-one unrelated drone samples resequenced for SNP detection and selection

Sample	Sex	Region, Country	Subspecies	Sequencing output (Gbp)	Average depth of coverage
1	male	Südbrookmerland, Germany	*carnica*	9.575	31.2
2	male	Bayrisch Gmain, Germany	*mellifera*	8.643	31.1
3	male	Burgoberbach, Germany	*mellifera*	12.613	46.4
4	male	Altshausen, Germany	*carnica*	11.128	40.4
5	male	Altshausen, Germany	*carnica*	11.992	43.9
6	male	Altshausen, Germany	*carnica*	13.199	51.5
7	male	Flintbek, Germany	*carnica*	12.035	44.5
8	male	Sankt Veit im Pongau, Austria	*mellifera*	13.364	52.1
9	male	Lütjensee, Germany	*carnica*	12.478	49
10	male	Kirchhain, Germany	*carnica*	10.762	41
11	male	Hamburg, Germany	*carnica*	10.463	38.8
12	male	Veghelde, Germany	*carnica*	10.55	37.8
13	male	Nettersheim, Germany	*carnica*	11.916	42.2
14	male	Tostedt, Germany	*carnica*	8.107	30.6
15	male	Schleiden, Germany	*carnica*	12.579	49.2
16	male	Wittlich, Germany	*carnica*	9.775	34.2
17	male	Tostedt, Germany	*carnica*	10.303	40.4
18	male	LIB, Germany	*carnica*	17.762	64.8
19	male	LIB, Germany	*carnica*	10.945	39.7
20	male	Ljubljana, Slovenia	*carnica*	13.812	50.7
21	male	Sendenhorst, Germany	*mellifera*	14.324	53.1
22	male	Frontenhausen, Germany	*carnica*	11.095	44.6
23	male	Frontenhausen, Germany	*carnica*	10.062	36.6
24	male	Ljubljana, Slovenia	*carnica*	15.616	61.8
25	male	Ljubljana, Slovenia	*carnica*	12.985	48.1
26	male	Ljubljana, Slovenia	*carnica*	11.29	32.6
27	male	Mayen, Germany	*carnica*	14.226	51.2
28	male	Mayen, Germany	*carnica*	19.266	68.8
29	male	Mayen, Germany	*carnica*	13.829	49.5
30	male	Azmoos, Switzerland	*mellifera*	12.301	42.3
31	male	Aufhausen, Germany	*carnica*	10.487	36.6
32	male	Aufhausen, Germany	*carnica*	20.719	71.4
33	male	Aufhausen, Germany	*carnica*	14.671	50
34	male	Hall in Tirol, Austria	*mellifera*	11.703	41.7
35	male	Vikesa, Norway	*mellifera*	12.161	46.4
36	male	Vikesa, Norway	*mellifera*	12.853	49.3
37	male	Vikesa, Norway	*mellifera*	14.313	52
38	male	Vikesa, Norway	*mellifera*	14.059	51.8
39	male	Hoppegarten, Germany	*carnica*	11.829	43.4
40	male	Crostau, Germany	*carnica*	11.033	33.8
41	male	Vitters, Switzerland	*mellifera*	11.556	39
42	male	Steinhagen, Germany	*mellifera*	12.529	47.2
43	male	Weißkirchen, Austria	*carnica*	10.565	41.5
44	male	Lunz am See, Austria	*carnica*	12.807	46.9
45	male	Lunz am See, Austria	*carnica*	7.202	27.5
46	male	Lunz am See, Austria	*carnica*	8.661	30.9
47	male	Münchsdorf, Germany	*carnica*	15.689	4.3
48	male	Werdau, Germany	*carnica*	12.744	48.8
49	male	Ljubljana, Slovenia	*carnica*	11.529	45.7
50	male	Ljubljana, Slovenia	*carnica*	12.54	43.9
51	male	Ljubljana, Slovenia	*carnica*	12.394	48.1
52	male	Ljubljana, Slovenia	*carnica*	10.926	42.9
53	male	Ljubljana, Slovenia	*carnica*	9.262	31.6
54	male	Ljubljana, Slovenia	*carnica*	14.031	46.6
55	male	Ljubljana, Slovenia	*carnica*	21.381	71.9
56	male	Ljubljana, Slovenia	*carnica*	9.909	38.7
57	male	Salvator, Austria	*carnica*	12.573	49.2
58	male	Hamminkeln, Germany	*carnica*	12.338	47.6
59	male	Lübbecke, Germany	*carnica*	7.347	25.8
60	male	Ihlow, Germany	*carnica*	8.481	31.1
61	male	Brandenburg, Germany	*carnica*	16.715	63

Appendix 1 - Jones et al.

Table S3. Candidate regions selected from the existing literature.

Dataset	Description	Type	Distance criteria (bp)	Data size	Citations
1	Varroa resistance and hygienic behavior related	Gene	300	5167	[1-12]
14	Subspecies related	Gene	300	109	[13,14]
16	Social immunity	Gene	300	53	[15]
17	Flight and fight	Gene	300	13	[16]
18	Sex determination related	Gene	300	16	[17,18]
22	Foraging and nest defense, relevant SNPs	SNP	300	1415	[19]
24	*Apis mellifera* gene list	Gene annotation	300	15314	[20]

Table S4. Number of candidate genes selected in signalling and immune pathways.

Pathway		Number of *A. mellifera* genes
Signalling	Notch	31
	JAK-STAT	335
Immune	Toll	37
	SUM	403

Table S5. Gene keywords searched in the NCBI database.

Keywords	Number of *A. mellifera* genes
olfact*	50
flight	7
longevity	2
resist*	48
forage*	6
heat/thermal*	104
Immun*	4
odor	186
P450	56
stress	32
defens*	19
dscam	2
Vitellogenin	6
ProPO	113
abacein	1
serine protease	102
thioredoxin peroxidase	5
apolipophorin	5
SUM	748

References

1 Arechavaleta-Velasco, M. E., Alcala-Escamilla, K., Robles-Rios, C., Tsuruda, J. M. & Hunt, G. J. Fine-scale linkage mapping reveals a small set of candidate genes influencing honey bee grooming behavior in response to Varroa mites. *PLoS One* **7**, e47269, doi:10.1371/journal.pone.0047269 (2012).

2 Behrens, D. *et al.* Three QTL in the honey bee Apis mellifera L. suppress reproduction of the parasitic mite Varroa destructor. *Ecol Evol* **1**, 451-458, doi:10.1002/ece3.17 (2011).

3 Boutin, S., Alburaki, M., Mercier, P. L., Giovenazzo, P. & Derome, N. Differential gene expression between hygienic and non-hygienic honeybee (Apis mellifera L.) hives. *BMC Genomics* **16**, 500, doi:10.1186/s12864-015-1714-y (2015).

4 Cardoen, D. *et al.* Differential proteomics in dequeened honeybee colonies reveals lower viral load in hemolymph of fertile worker bees. *PLoS One* **6**, e20043, doi:10.1371/journal.pone.0020043 (2011).

5 Gempe, T., Stach, S., Bienefeld, K., Otte, M. & Beye, M. Behavioral and molecular studies of quantitative differences in hygienic behavior in honeybees. *BMC Res Notes* **9**, 474, doi:10.1186/s13104-016-2269-y (2016).

6 Gibson, J. D., Arechavaleta-Velasco, M. E., Tsuruda, J. M. & Hunt, G. J. Biased Allele Expression and Aggression in Hybrid Honeybees may be Influenced by Inappropriate Nuclear-Cytoplasmic Signaling. *Front Genet* **6**, 343, doi:10.3389/fgene.2015.00343 (2015).

7 Gregorc, A., Evans, J. D., Scharf, M. & Ellis, J. D. Gene expression in honey bee (Apis mellifera) larvae exposed to pesticides and Varroa mites (Varroa destructor). *J Insect Physiol* **58**, 1042-1049, doi:10.1016/j.jinsphys.2012.03.015 (2012).

8 Mondet, F. *et al.* Antennae hold a key to Varroa-sensitive hygiene behaviour in honey bees. *Sci Rep* **5**, 10454, doi:10.1038/srep10454 (2015).

9 Navajas, M. *et al.* Differential gene expression of the honey bee Apis mellifera associated with Varroa destructor infection. *BMC Genomics* **9**, 301, doi:10.1186/1471-2164-9-301 (2008).

10 Oxley, P. R., Spivak, M. & Oldroyd, B. P. Six quantitative trait loci influence task thresholds for hygienic behaviour in honeybees (Apis mellifera). *Mol Ecol* **19**, 1452-1461, doi:10.1111/j.1365-294X.2010.04569.x (2010).

11 Tsuruda, J. M., Harris, J. W., Bourgeois, L., Danka, R. G. & Hunt, G. J. High-resolution linkage analyses to identify genes that influence Varroa sensitive hygiene behavior in honey bees. *PLoS One* **7**, e48276, doi:10.1371/journal.pone.0048276 (2012).

12 Hu, H. *et al.* Proteome Analysis of the Hemolymph, Mushroom Body, and Antenna Provides Novel Insight into Honeybee Resistance against Varroa Infestation. *J Proteome Res* **15**, 2841-2854, doi:10.1021/acs.jproteome.6b00423 (2016).

13 Wallberg, A. *et al.* A worldwide survey of genome sequence variation provides insight into the evolutionary history of the honeybee Apis mellifera. *Nature Genet* **46**, 1081-1088, doi:10.1038/ng.3077 (2014).

14 Zhang, Z. *et al.* Transcriptome Analysis of Female and Male Xiphophorus maculatus Jp 163 A. *PLoS ONE* **6**, e18379, doi:10.1371/journal.pone.0018379.t001 (2011).

15 Le Conte, Y. *et al.* Social immunity in honeybees (Apis mellifera): transcriptome analysis of varroa-hygienic behaviour. *Insect Mol Biol* **20**, 399-408, doi:10.1111/j.1365-2583.2011.01074.x (2011).

16 Hunt, G. J. Flight and fight: a comparative view of the neurophysiology and genetics of honey bee defensive behavior. *J Insect Physiol* **53**, 399-410, doi:10.1016/j.jinsphys.2007.01.010 (2007).

17 Beye, M. The dice of fate: the csd gene and how its allelic composition regulates sexual development in the honey bee, Apis mellifera. *Bioessays* **26**, 1131-1139, doi:10.1002/bies.20098 (2004).

18 Miyakawa, M. O. & Mikheyev, A. S. QTL Mapping of Sex Determination Loci Supports an Ancient Pathway in Ants and Honey Bees. *PLoS Genet* **11**, e1005656, doi:10.1371/journal.pgen.1005656 (2015).

19 Hunt, G. J. *et al.* Behavioral genomics of honeybee foraging and nest defense. *Naturwissenschaften* **94**, 247-267, doi:10.1007/s00114-006-0183-1 (2007).

20 Christine G Elsik *et al.* Finding the missing honey bee genes: lessons learned from a genome upgrade. *BMC Genomics* **15**, 86 (2014).

Table S6. SNP statistics for 2734 bees collected across Germany, Austria, the Netherlands and Switzerland (beebreed.eu).

Total SNPs included	SNPs <90% call rate	SNPs <5% / <1% MAF	SNPs < HWE ($\boldsymbol{P < 10^{-7}}$)	Total SNPs to be removed
103270	10450	30813 / 16566	1067	42330 / 28154
